# The Thirty-sixth Annual Meeting of the Michigan Dental Association

**Published:** 1892-01

**Authors:** N.S. Hoff

**Affiliations:** Ann Arbor, Mich.


### THE DENTAL REGISTER.

Vol. XLVI.] JANUARY, 1892. [No. 1.

### COMMUNICATIONS.

### The Thirty-sixth Annual Meeting of the Michigan Dental Association.

Sault Ste. Marie, Mich., August 18, 1891.

Reported by N. S. Hoff, D.D.S., Ann Arbor, Mich.

The association meetings were held in the Court-room of the Court House. The first session was called to order at 10½ o'clock by President Dr. H. K. Lathrop.

Rev. P. T. Rowe was present and invoked the Divine blessing upon the meeting.

The Hon. Geo. M. Brown, mayor of the city was introduced and made an address of welcome, complimenting the members of the association and the profession generally. The citizens of Sault Ste. Marie were highly honored to have the association meet in their city, and as its representative, Mr. Brown desired most cordially to welcome the members individually and the association to all the privileges and hospitality of the city.

The President thanked Mr. Brown for his cordial welcome, and read his annual address.

PRESIDENT'S ADDRESS.

For the honor which has been your pleasure to confer upon me to be your presiding officer for this meeting, I return you my most sincere thanks. I am not insensible to the importance of the position to which you have called me, and I have grave fears of my ability to properly fill the position, for I have little or no education in parliamentary law. But in discharging the duties which are incumbent upon me, I shall endeavor to serve you to the best of my knowledge and ability. I hope and trust, and indeed I am sure that I shall be fully sustained and aided in that labor by every member present.

We meet here to-day under the most favorable auspices, harmonious and prosperous, so let our meeting and relations ever remain to our mutual benefit. Dentistry scarcely a century old, has risen from its humble origin and become a highly respectable power for good throughout the world. At first it was confined to and practiced by a few ingenious and self-taught workmen who made their own instruments and materials and did the best they could under the circumstances and conditions of the times. Now by concerted action, by science and association, we have arrived at a highly commendable condition of attainment in the beauty and usefulness of our manipulations.

The success that has attended our efforts for improvement is a matter of which we may well be proud; for dentistry has kept pace with the movements of the age and now claims to be a profession the peer of any other.

In our efforts to raise our profession to its present exalted position, we have done nobly for ourselves and for humanity. Our object has been, and I hope will continue to be, to attain the greatest usefulness and perfection in an honored calling.

All societies and associations are composed of members enough it is hoped to accomplish the particular object for which they were organized. In the less thickly populated sections of the country such organizations are seldom found, and the want of companionship and society is sadly felt; but in large cities and thickly settled towns, such oranizations are much more easily formed and maintained.

The members of any profession widely separated, find it very difficult to keep up with the march of their chosen calling, and to obtain that skill which will give them confidence in their ability for success and to meet on equal terms their more favored brothers. Such isolated practitioners of dentistry are apt to fall into methods and customs of their own, which are very liable to be full of imperfections. They are apt to recognize no code of ethics or practice and become a law unto themselves, and it requires a great amount of courage on the part of any one to question their lack of attainments.

The dentist should be a man of skill, sound judgment, and above all, honest. Now to give these attainments to the members of our profession, the colleges, societies and associations, have done very much, but it seems to me that still other organizations could do still more ; just how or in what way I have only a vague idea in my own mind. I was impressed by the remarks of Dr. W. H. Richards, of Knoxville, Tenn., before the Southern Dental Association. He favored a permanent place of meeting that could be called a home, have a library and museum, and he said the advantages to be gained by this course were, that the sessions could be extended to such time as would give ample opportunity to carry through in detail all clinics, lectures or mechanical procedures, and have lectures on dentistry, and so much of the collateral sciences as pertains thereto, thus year by year raising the standard of dentistry and offering a post-graduate course to all members of the association. Now, gentlemen, I know of no better place to put such a thing to a practical demonstration than right here in our great State of Michigan, and although I have had very little talk with any one on the subject, I doubt if we would have any obstacles put in our way, of using lecture rooms, laboratories, library and museum of the Dental Department of the University of Michigan.

Ann Arbor is a beautiful little city, and a more pleasant place could hardly be found to spend a few days during the summer, when the regular departments of the university were not in session. This may not impress you all as it has me, but I offer it only as a suggestion, but it seems to me a step in the right direction. I hope you will all think about it, and if any of you think you have a better idea, tell us about it and I am sure we shall be able to devise some scheme that will accomplish our purpose.

*Dr. J. Taft.*—In these days the greatest and best things are accomplished, not by individuals alone, while it is generally the case that some one mind is responsible for the first idea or conception, most great and important enterprises are the result of combinations of kindred minds. This will be no where better illustrated than in the wonderful progress of dental art and science; in fact this associated effort has resulted in the exaltation of dentistry from mere handicraft to a scientific profession. It will be profitable to recall the influences for good of this association in this State, to enforce the thought that associated effort is good. It was because this association asked the legislature of the State to protect its people from charlatans and tyros that we now have the excellent laws in this State, protecting the qualified practitioner as well as the innocent public.

This association also, by concerted action succeeded in having established in connection with the great State University a department where dental science should be taught. And it ought to be the desire of every member of this society to most heartily concur in the suggestion of the president in regard to the establishment of a permanent home for the association, where a library, museum and laboratories should be available for any sort of study, or investigation that would provide better practitioners, or give opportunities for advanced thinkers to carry out experiments, which it would be impracticable for individuals to provide for themselves. No better work it seems could engage the attention of this meeting than to put this matter in the way of accomplishment.

By vote of the association the president was instructed to appoint a committee to consider the practicability and advisability of establishing a permanent place of meeting for the association. This committee, appointed since the meeting, is complete, as follows: J. Taft, N. S. Hoff, W. A. Dorland, J. Ward House, Wm. Cleland.

The chairman of none of the regular committees being present, no reports were made.

The afternoon of the first day was devoted to clinics. Dr. W. A. Dorland, of Grand Rapids, exhibited a combination "Plate and Bridge" which he had just constructed for a patient. It consisted of a small gold plate swaged to cover only the alveolar ridge of the upper jaw, except the third molar tooth on each side, which was covered with a collar having parallel sides and a flat cap, the tooth having previously been dressed to allow a nice adjustment. A collar clasp or band was made to fit around the first collar accurately and of sufficient width and strength to resist the movements of mastication. A cap with cusps was then soldered on top this band, making a crown, and this crown was then soldered to the gold plate. The idea being that the gold plate would have an advantage because of the limited amount of mucous membrane covered, and also because of its secure attachment by means of the accurate fitting crown to the cylindrical ferrule. This ferrule being previously cemented to the natural tooth—in this way a removable bridge is secured capable of being manipulated at will by the patient for cleanliness, etc., and a degree of stability secured which is impracticable with the ordinary plate, and the whole fixture presenting none of the objectionable features of permanently attached bridge-work. The regular "bridge dummies" with porcelain face and metallic caps were used as teeth and so soldered to the plate as to secure cleanliness and the largest mastication surface.

The doctor also exhibited a lower denture in process of construction involving the same ideas. The work was beautifully and artistically done and showed an amount of patience and skill that is highly commendable.

*Dr. E. R. Jackson*, of Grand Rapids, crowned a second upper bicuspid tooth with a gold Richmond crown, and attached a bicuspid dummy with a porcelain face and metallic cap to fill the space caused by the loss of the first bicuspid. This was a successful operation and readily accomplished in a very satisfactory manner, making a sightly, cleanly and useful substitute for the lost tooth. There were no special features in the doctor's methods except perhaps his method of stamping the caps for his crowns and dummies, which he obtains by stamping from solid buttons or pieces of thick gold by means of steel stamps and counter-dies of his own construction.

EVENING SESSION.

The meeting was called to order at 7:30 P. M. by President Lathrop and the minutes of the previous session read and approved.

*Dr. Isaac Douglas* read a paper entitled, Hints from the Office and Laboratory. If we would command the respect due to our profession we must attend carefully to the most common-place details of our work. Whether a patient does or does not have a glimmering of the higher attainments of a dentist he quickly takes in the ordinary arrangements of the office and habits of the operator, and he has a right to require not only general neatness, but special cleanliness.

I have seen in dental offices sights which made me blush for the brotherhood.

A dentist's hands, in particular, should not only look clean, but should *be* clean and *smell* clean, and perfect cleanliness is not likely to be attained without suitable conveniences. The neatest and most handy method of washing the hands is from a running stream. It takes less water when that is an object; and is much quicker, which is always an advantage. It is wholly unobjectionable. Those who are not connected with water-works will be well repaid for arranging a tank, even if no larger than a common water pail.

It would not require a very fastidious patient to become utterly disgusted with a dentist who would put his hands into four successive mouths, and back again into the first mouth, without any attempt at cleansing except to wipe them on a filthy napkin on the head-rest of the chair. Nor would his disgust be less, were he to see the filthy water, and the filthier wash-bowl where he finally washed his hands and the much-used towel where he wiped them.

If any one thinks this picture overdrawn it is only because his observation has been very limited in this direction.

Even though supposed to be clean the dentist's hands should always be washed just before commencing to work for a patient, that the latter may know their condition from their coolness and moisture, and may experience a feeling of comfort.

Tact and suavity are needful accomplishments when working for adults, and indispensable for children. In case of emergency the operator should be quick to invent a way to bridge it over; and when practicable should conceal his dilemma from the patient, but not deceive.

A few days ago I was putting in the last one of a large number of fillings. The tooth was a left upper lateral incisor. The anterior proximate side had been filed away before coming to me. After removing what had been softened by decay, the cavity was extremely shallow, the dentine being opaque further toward the pulp. When the cavity was three-fourths filled I broke through to the pulp; this being the first case of the kind in over forty years' practice. I revealed my dismay by the expression, "Oh, pshaw." The secret once out, I explained to the patient who remarked that it did not hurt. Having removed the filling and putting a sharp drill into the engine, I went to the pulp from the highest portion of the cavity. As soon as the drill struck the pulp I put in some crystals of cocaine, rested five minutes, then resumed with the drill until an opening was made sufficient to introduce a barbed broach. Put in some more cocaine, rested ten minutes, then with the broach removed the pulp entire. The patient then remarked that she had not suffered much. I then washed out and filled the root canal, and filled the crown cavity. The patient seemed more delighted with the results than if the accident had not occurred, remarking that the operation was not as painful as the separation of teeth with the separator.

To keep dental cavities dry while filling, it is necessary to resort to a great many expedients, according to the situation and other circumstances. With many small cavities a piece of spunk or string, or a roll of paper, crowded under the free margin of the gums, will serve the purpose admirably; cut the gums loose a little way if necessary.

Below a labial cavity in a lower cuspid the gums and alveolar border had receded so that the cavity extended nearly one-eighth of an inch below the margin of the alveolar border upon the opposite side of the tooth. This is a very perplexing case to fill with gold. Take a piece of hard wood, shape it like a wood-carver's gouge, only let each corner project that it may pass in more or less between the teeth, fitting it to the position on the tooth. Before applying the dam saturate a thin piece of spunk with a 20 per cent. solution of cocaine and lay it on the gums for five or ten minutes; do not have too much of the solution lest it mix with the saliva. Now apply the rubber dam; also the ligature; tie loosely for the present. Pull the rubber and ligature-knot downward in front of the tooth so as to expose the entire cavity and margin of the gums above the rubber. Place the stick in position and hold the rubber below the cavity firmly; let loose the rubber and with a thin instrument carefully work the rubber to its place. In this an assistant may be of great service by tightening the ligature gently while holding the stick firmly in place with the left hand. The cavity may now be prepared and filled entirely with the right hand.

There are many cavities where we can not put in a first-class gold filling, but can put in an excellent filling of amalgam; and without question this is much better than a poor gold one, for nothing will preserve a tooth better than the best amalgam filling.

Take a case of a left lower wisdom tooth with a large crown filling of amalgam, which has done service for eleven years, yet is still in perfect condition. A large cavity has formed in the posterior buccal side; three fourths of the posterior side and one-half of the buccal side are gone nearly to the margin of the gums, and when prepared, to the gum and below. Of course the gum is fretted and cut more or less by the excavators, and bleeding slightly. Wash out the cavity, dry it with paper or spunk, and fill with dry absorbent cotton, allowing the cotton to extend over the gums. Dip a pellet of cotton in alcohol and touch that in the tooth to saturate it. Twist quite hard a piece of rather course ligature-twine, double and twist hard again, wax well and pass through the flame of a lamp to melt in the wax. Remove the cotton from the tooth; there is now no hemorrhage. Pass the twine around the tooth. If the second molar is too close to admit so large a cord between the crowns, run a piece of floss silk between the strands at the doubled end of the twine, pass the doubled floss between the crowns, and, by it, draw the cord through between the necks of the teeth. Tie the twine around the tooth by a surgeon's knot on the lingual side. Do not draw it tightly at first as it will lie across the cavity, but carry this part backward and push it down between the neck of the tooth and the gums. This excites slight hemorrhage which can be stopped as before. The pulp of this tooth was destroyed about one year ago, and the posterior cavity filled—or rather, stuffed by a tooth-carpenter. After removing the crown filling, and after properly preparing and filling the root canals, again fill the cavity with cotton and saturate with alcohol, as before, yet not pressing it on the gums very hard this time, lest the removal of the pressure cause the blood to flow where the gums may have been cut. Prepare the amalgam and press it pretty dry. Now, either fix a pad of bibulous paper, sponge, or fold the corner of a napkin to press against the parotid duct; twist the napkin aud lay it between the tongue and jaw. Tip the head well to the right, remove the cotton, take up the excess of saliva, dry all with a warm air syringe, and the cavity is ready for the filling.

Take rather a large piece of amalgam at first, so as to cover the part next to the gum at once, pressing it down firmly. The more force used in packing the filling the better the prospect of its lasting for many years. After thoroughly condensing the filling dress it to shape, being careful that it does not project beyond the margin of the neck of the tooth.

Suppose a case of two approximate cavities in upper molars reaching far above the enamel of the tooth; the gums having receded. It is impossible to make the dam stay above the cavities, but by putting a string around each tooth, as described heretofore, the two strings nearly or quite fill the space between the two teeth above the cavities. After preparing and packing and again drying, as in the other case, fill as one cavity, letting the first piece of filling be large enough to cover the upper portion of both cavities at once. After they are well filled let the patient close the mouth and grind down on them well, provided the lateral walls are intact, so there is no danger of breaking down the filling. Then cut them apart and trim into that shape which will be the most durable and comfortable for the patient.

For gold fillings in those small cavities sometimes called pin-holes, first drill out the cavity round to remove the decay. Then with a small, round bur very slightly undercut on one or two sides, just enough to insure the filling against turning while burnishing. With a bur a few sizes larger than the first bevel the edges of the walls slightly, and after washing and drying the cavity is ready to fill.

To prepare the filling, have a piece of bone, a tooth-brush handle will do, with holes graded to correspond with the engine bur. With a tapering square rimmer enlarge these holes from one side so that they will be tapering most of the way through. Make a cylinder larger than the cavity when rolled snugly between thumb and finger. Force this through a number of successive holes until it will just fit the round part of the cavity. When the cavity is clean and dry insert this prepared plug. If all is perfectly done one can hear it squeak as it is forced through the round part of the cavity. Drive it home with plugger and mallet, and there is the most perfect filling that can possibly be put in such a cavity. In this way I have filled such cavities no larger than No. 00 burr. I have put in six of this kind, besides two other fillings in the grinding surface of one molar.

This method also works well in case of quite large tin fillings in the posterior surface of bicuspids and molars when there was such a profuse flow of saliva that it was just impossible to keep the cavities dry long enough to fill them in the ordinary way. Of course the tooth back of the one filled must be missing to fill in this way.

In malleting sore teeth hold a piece of block tin, lead or hard wood against the opposite side.

To prevent the pain from the application of oxyphosphate near the pulp, touch with a thin solution of gutta percha in chloroform; over this lay a piece of No. 8 tin foil, or a flattened pellet of gold. Then put in the oxyphosphate.

Exposed pulps may be capped in a similar way by adding a small amount of oil of cloves to the gutta percha solution. If the pulp be wounded, stop the hemorrhage with chloroform and take away the clot so made before capping.

For pyorrhœa alveolaris, try a freshly prepared saturated solution of sulphate of copper. To apply, take a hard wood stick the size of a match, cut one end down to the thickness of thick writing paper, roughen the edges, twist on a very little cotton, dip into the solution and pass into the pockets after pretty well removing the moisture with a napkin or bibulous paper. This works like magic.

To prevent dark work in rubber work, and to prevent breaking blocks, grind and match the blocks together accurately. Bevel the corners toward the ridge slightly. After the piece is flasked put in warm water until warmed through. Separate as soon as warm enough. Peel out the body of the wax; wash out the rest of the wax with boiling water from one foot above. With a thin instrument press a No. 50 cotton thread into the v-shaped groove letting the ends of the thread dip into the plaster on both sides, or at the ends. Cut a groove in the plaster next to the half of the flask that contains the model, to receive the surplus rubber. Make waste-gates every one-fourth inch all the way around, making them the shape and size of one half a No. 9 iron wire, or even larger. Be careful not to put in so much rubber that the excess will fill the trench. Have your flasks hot and give the rubber plenty of time to move; it will not move quickly under pressure except by too great force.

It is sometimes the case, particularly with a partial upper denture, that the plate will be as snug as desirable part of the time, but sometimes very loose. To avoid this, before casting your model, make the grooves in the impression caused by the rugæ of the mouth much larger, as in some mouths these vary in size at intervals.

To secure the blessings of your patients polish the gingival and palatal surfaces of all rubber plates.

### REFLEX NERVOUS ACTION.

Mrs. P., aged 32, was taken with pain in back of head and neck. A physician pronounced it neuralgia, and treated her several months, the patient meantime growing worse. A third M.D. called it spinal affection, and doctored her the remainder of two years. She then became insane. Then the physician advised her husband to take her to an asylum, but they took care of her at home. After she had been insane two weeks, she became rational one morning, and remarked, "I am going to Romeo to have my tooth pulled." This was the first complaint that had been made about the tooth. I was called to the house and extracted a right lower wisdom tooth with an abscess attached on the apex of the root as large as a green pea. This abscess had discharged through the tooth just enough to prevent its breaking elsewhere, but not enough to relieve the pressure; and the pain was reflected, first to the back of the neck and back of the head, and finally to the waist and elbows. The removal of the tooth ended the whole trouble, and twenty years after, the symptoms had not returned.

Mr. B., seated in the chair, preparatory to having a tooth extracted, said: "I have had pain in my right elbow for three months; some of the time it has been swollen and lame, so that I could not feed myself with my right hand. I have not had a bit of pain in my tooth till this morning. Now I want to know if there is a nerve running from my elbow to my tooth as a part of the time the pain is in my tooth and a part of the time in my elbow?"

Mrs. S., aged 29, after suffering with a tooth for several days, came nine miles to have it extracted. It was evident at a glance that she was suffering from chorea. After three or four minutes she became quiet, and her friend remarked, "she has been in this condition for three days; please extract her tooth as soon as you can, and I will tell you about it afterward." As I put the forceps to her mouth, the spasm came on again. As soon as it subsided, I extracted the tooth. An expression of relief came over her face at once. Her friend then remarked, that the tooth would ache three or four minutes; the moment the pain left the tooth, she would commence to whip her hands together, and keep it up till the pain returned to the tooth. As soon as the tooth commenced to ache, the hands became quiet. This had gone on for three days, and her hands had become so sore that when she struck them together she would cry out like a child.

Mrs. A., aged 49, came for consultation about her left eye. It had pained her for two or three weeks. Examination revealed the fact that the pupil was drawn out of normal form, the larger diameter being double that of the lesser. Yet no inflammation was perceptible. Although she declared she had no trouble with the teeth, yet examination proved that the crown of the upper sixth-year molar was two-thirds wasted away, and the pulp very largely exposed. She would not be persuaded to have the tooth removed; and two weeks from that time, a darkness came over that eye, and she never recovered the use of it. Had the tooth been extracted in time, undoubtedly the eye would have been relieved.

DISCUSSION.

*Dr. J. Taft.*—There are many good points in Dr. Douglas paper that we might discuss with profit to all present. But the matter of personal cleanliness it seems to me, is one that ought to be of special interest to every dentist, for it is a question of the utmost importance and means oftentimes success or failure. Some people are naturally careless and uncleanly, and can't appreciate the nice distinction between being clean and orderly, or the reverse. Others again are unclean because they allow business to interfere with order and the necessary attention to decent toilet preparations. The man who is unclean because he knows no better or it is a part of his make up, may be excused perhaps more than one who allows the demands of business to so encroach upon the requirements of decency as to neglect to keep himself and his surroundings acceptable to his patients. If you have no time to keep your office clean, employ an assistant to look after this and perhaps relieve you of some other unnecessary labor, in order that time may be had in which to make yourself acceptable to your patients. A carefully trained office assistant is a necessity and a blessing as well as a luxury.

Personal health which makes one unacceptable or disagreeable, demands careful attention. Bad breath is especially disagreeable and can generally be avoided. When a disagreeable breath results from objectionable and filthy habits, it ought not to be tolerated for a moment by any self-respecting person.

The doctor related an instance of where a well-known dentist was called upon by a colored girl for some dental treatment, and because of the careful toilet preparation he made before and during the treatment, he so impressed the girl that she related the fact in the wealthy home where she lived, and as a result a large and wealthy connection became the dentist's patrons.

An appeal to the mercenary inducement is not the only or highest motive to urge to induce dentists to become cleanly, but the reaction upon the man himself will be of incalculable value, lifting him to a higher plane of self respect and morality.

*Dr. Douglas* thought the dentist's cuspidore should receive more attention than it generally does. He recommended washing thoroughly and then smoking them over burnt paper. Light a ball of paper and hold over it the cuspidore until it is hot, and then set it down over the burning paper and leave over night.

*Dr. N. S. Hoff.*—The matter of personal cleanliness is one that merits careful consideration as it is of the greatest importance, but cleanliness of operating instruments and appliances is one that is equally important if it really does not transcend all other sanitary considerations. The invariable presence in every condition of the mouth requiring the interference of the dentist, of disease inoculating influence, which necessarily must contaminate every instrument used, renders cleanliness in respect to instruments and appliances of the utmost importance. Every dentist should provide himself with simple and sufficient means for safely sterilizing every instrument used in connection with operations upon the teeth and gums; and no instrument once used should be used on another patient, and generally not on another tooth in the same mouth without its having been cleansed and sterilized. This is particularly true in regard to excavators, burs, scalers, nerve canal instruments, and rubber-dam clamps, and applies with more or less force to other appliances.

There is a statement in the paper in regard to the efficacy of amalgam as a filling material that I cannot agree with, and I don't think it is the accepted belief of the profession. The Doctor says, "Nothing will preserve a tooth better than the best amalgam fillings." Amalgam as a filling material is notoriously faulty and unreliable. While it is true that occasionally an amalgam filling will have apparently preserved a tooth from the further encroachment of caries, it does not always follow that any particular skill or care was employed by the operator in making it. In most of these cases good and sufficient reasons may be given for the favorable result. The most skillful and conscientious operators have given amalgam more than a fair test; they have earnestly desired to demonstrate it a reliable filling material, and used it long after it had proved itself to them, by its continued failures, unworthy of confidence. It is not just to pick out the isolated cases where amalgam has apparently proved itself a reliable material, and draw the conclusion that the best amalgam filling will save teeth as long as any other filling material. The statement is misleading and may be looked upon as an endorsement of the material. At the same time it is a reflection upon the manipulative attainments of some of the best men in the profession. Moreover we don't think it is true that the number of good amalgam fillings will average proportionately with the number of good gold fillings.

*Dr. Saunders.*—I have often heard that amalgam made as good fillings as gold, but I do not believe it. My experience does not demonstrate it. It is sometimes a difficult matter to determine that one filling is going to save a tooth and another will not; or that one filling is better than another when both are apparently accomplishing the object for which they were made. My reason for not believing in amalgam is not based on the fact that it always fails, but that it is more likely to fail on the average than gold. I do not think I fail in manipulating the material; I take all the necessary precautions I do with other materials; I work it dry and use all other necessary precautions. It seems to me that the fact that we are continually striving to learn to use this material in such a way that it may not prove a failure is good evidence that it is not as good a material as gold for filling teeth. I believe gold is the most reliable material for filling teeth. But it must be used skillfully, and judgment must be exercised in the selection of cases; that is, the nature of the tooth structure to be operated upon, extent of caries, difficulties of approach, etc. While I believe gold is the best filling material I most decidedly hold that amalgam has a place as well as gold, cement, tin and gutta-percha. It should be our constant aim to use the material that judgment based upon science and experience has demonstrated to be the one adopted to the particular case in hand.

*Dr. Sweetnam.*—Much of the cause of failure in fillings is due to the fact that due care is not exercised in the preparation of cavities. Thin or weak walls should be cut away. Neither do I believe in making six fillings in the crown of a molar tooth, the fissures should be cut out and the cavities made into one. The paper suggests a treatment for capping and saving a pulp which has been bleeding; by coagulating the blood, removing the clot and then capping the nerve. I can't save pulps that have reached this degree of exposure and don't consider it practicable to undertake it. The paper also suggests manufacturing a cylinder and driving it into simple crown cavities. I don't think a filling made in this way would prove at all reliable, because it could not be made to conform to the cavity walls.

*Dr. Douglas.*—I think my experience ought to justify me in maintaining, or at least expressing the opinion, that amalgam when properly manipulated will preserve the teeth. I have practiced in one place thirty years, and have seen fillings recently that I put in many years ago, and I have only recently seen a filling made in the first years of my practice that was preserving the tooth perfectly. I think good amalgam skillfully manipulated will preserve the teeth as well as any other material.

*Dr. J. Taft.*—It is surprising to me that any one who has had such success in the use of amalgam, as Dr. Douglas, should ever use anything else. While it may be true that amalgam is a safe and reliable filling material in Dr. Douglas' hands, it has woefully fallen short of giving satisfactory results in the hands of most operators. One great difficulty with this material is that it encourages faulty and careless manipulation, there is nothing in its manipulation to call out the highest ability of the operator, and the very ease with which it is manipulated is a source of evil in more ways than one. The material itself has inherent, harmful and bad qualities which ought to debar it from use. Of its practical use, perhaps I ought not to say anything, for I have never yet inserted an amalgam filling in any patient's tooth; but from what I have seen of those made by other operators I don't think I shall begin now.

*Dr. Douglas.*—I use amalgam successfully and I can't think that others fail for any other reason than that of faulty manipulation. I am just as particular to prepare the cavity for an amalgam as for a gold filling. I work the amalgam dry, press it in strong linen or buckskin—never use chamois skin, as the fibres of the chamois will imbed themselves in the material and prevent its perfect crystalization—when all the mercury has been squeezed out that is possible, it will still contain sufficient to make it pack readily. I always wash the material in alcohol.

*Dr. Hoff.*—I do not think the trouble is in the manipulation at all. I have used all of the best and most expensive amalgams made, and have adopted every suggestion that seemed to have any reason in it, and the sum total has been, in my hands, failure. I don't think the fault is in me altogether, but am compelled to conclude that a material demanding such nicety in manipulation, does not compensate adequately in the results secured.

The Secretary read a paper written by Dr. G. E. Corbin, entitled.

A STRANGE CONDITION. DID ARSENIC PRODUCE IT?

A few months since, a small woman with fairly good health presented herself for attention.

I found that the crowns of eight molars had been lost by decay to such an extent that I extracted their roots separately, and easily.

Two incisor and one canine tooth of the lower jaw, were crownless and pulpless. Three bicuspid and two incisor teeth of the upper jaw were also crownless and pulpless. These eight teeth I have since supplied with artificial crowns.

The crowns of the other two upper incisor teeth were pulpless, and nearly half destroyed by decay, but their contours have been restored with annealed gold under the mallet.

The crown of the fourth upper bicuspid was pulpless, and half destroyed by decay. It is now filled with oxyphosphate of zinc.

My astonishment over this pulpless condition was greatly increased when informed by the patient that she never had toothache in her life! Half the crown of the right inferior first bicuspid was restored with gold foil.

A large gold filling was inserted in right superior first molar. In preparing the last two teeth mentioned, excavations in the dentine could be felt, but produced no decided pain. Gold fillings were inserted in several other teeth where there was no sensation in the dentine whatever, but as the teeth were firmly set, of good color, and surrounded by healthful tissues, I did not open into their pulp chambers. Another excuse for not doing so, if apology seems to be necessary, that with all of these bad teeth, she has never had an alveolar abscess! My curiosity was so great as to induce me to institute a careful inquiry concerning the woman's history and habits. In October, 1880, her condition was such as to cause her to consult a physician, so called. "The Doctor" prescribed "Fowler's solution." This the patient took in "in six drop doses," three doses each day as directed, without any knowledge or suspicion of the *nature* of the solution.

Getting no better, "the doctor" was again consulted, and repeated his prescription. After weeks of perseverance in the use of the medicine, new and troublesome symptoms arose.

The number of the prescription being on the bottle, the medicine was procured of the druggist and taken with punctuality for months; the patiently constantly growing worse, until, for the last six weeks of its use she was confined to her bed, in a darkened room, with inflammation of the conjunctivæ, suffusion of eyes, feet and ankles swollen, general anasarca in fact, and stomach too irritable to retain any solid food. In short, she was dangerously ill from slow arsenical poisoning, extending over a period of eight months; from October, 1880, to June 1881.

She reports that her teeth were all sound at that time—ten years ago.

As there is no effect without a cause, the question for consideration is to determine the cause of such extensive devitalization.

The fact that arsenic is made use of for the destruction of pulps, by virtue of its caustic effect when locally applied, will throw no light on this case. For months the whole system was saturated with arsenic and the vital powers greatly depressed in consequence. The usual symptoms of slow arsenical poisoning were present. That the coats of the blood vessels were enfeebled, collapsed, was sufficiently proved by the general anasarca. The congestion of the capillary blood vessels and effusion into the cellular tissues about the eyes, in this as in other cases, was well marked and one of the earliest symptoms. A similar congestion in, and effusion from the capillary vessels of the pulps of the teeth must have taken place. From the intolerance of food and general functional inaction the whole system suffered from deficient nutrition.

The privation of nutrition from the pulps may have been greater than from any other portion of the system. Both anatomical and mechanical causes can be assigned for this opinion.

The congested condition of the capillaries may have been such as to greatly restrict and finally entirely intercept the flow of nutriment through the small and unyielding apical foramina. The arsenical solution was taken for eight months, and the process of destruction of the pulps may have continued for many months, or even years, longer. Indeed, the diminished sensibility of the dentine in teeth recently filled, indicates that the process of devitalization is still at work.

This slowness of destruction has given time for the disposition of the products of decomposition through the process of absorption and elimination, as the result of increased glandular activity. Certainly this very strange condition had a cause. If the above history and suggestions throw no light upon it, will some one, better qualified, give us the true solution?

DISCUSSION.

*Dr. Douglas.*—As I listened to the description of the symptoms as described in the paper, I said to myself how true and perfectly are the symptoms of arsenical poison here described, corresponding exactly with the homœopathic materia medica description of the manifestation of this drug. I have knowledge of a case in which the pulps of a child's teeth were destroyed by a similar use of this drug.

*Dr. J. Taft.*—The physician or dentist ought to take into consideration the susceptibility of the patient when about to administer drugs that are capable of producing by over dosage, harmful functional disturbances. And as these conditions are not always readily recognized it is a wise precautionary measure to begin the administration with the minimum dose and, inform the patient of the nature of the medicine used and the danger of prolonging its use, or over dosing. Had such precaution been taken in the case related by Dr. Corbin much suffering and injury would have been spared.

Serious mischief has often occurred from the use of arsenious acid applied topically. An unnecessarily large dose, or careless use of a proper quantity has resulted in serious injury. It is always good practice to be on the safe side when using poisonous drugs; therefore begin with small doses and increase as the system will tolerate and the circumstances indicate.

*Dr. Jos. Lathrop.*—Why was it, Dr. Taft, in this case related by Dr. Corbin, that the pulps were destroyed and other tissues were not devitalized?

*Dr. Taft.*—It is probably due to the fact that the inflammation set up in the system generally, affected the pulps, and these organs being inclosed in solid, unyielding walls, the capillaries become clogged and nutrition being cut off, death ensued from starvation.

[Dr. Corbin in the paper expresses surprise that no alveolar abscess from any of these teeth had taken place. This can easily be accounted for by the fact that these pulps died because of the inflammation consequent upon the administration of the arsenic systemically, consequently no micro-organism could be present to produce putrefaction, which would be the case if there had been an external opening from caries to the pulps. The absence of toothache can also be accounted for from the fact that the presence of the arsenic in the pulp tissue had narcotized the nerve endings to such a degree as to incapacitate them for conveying impression in the normal manner, and the death of the nerve cells and atrophy of the other tissues without any putrefactive change produced no inflammation in the contiguous tissues, which were not destroyed by the general inflammatory conditions. It would be interesting to know whether the peridental membrane was at any time unduly sensitive.—HOFF].

*Dr. Douglas.*—I put some arsenical paste in the tooth of a boy thirteen years old and told him to come back to me in twenty-four hours. It was ten days before I again saw him, and I extracted the tooth at once, because the blood corpuscles had become disorganized and broken up, and passed into the tubuli of the dentine staining the whole tooth to a bright pink color. The utmost caution should be observed in making applications of arsenic to children's teeth, and in no case should it be permitted to remain there a long time.

The subject was passed and the meeting adjourned to meet Wednesday morning at 9:30 A. M.

WEDNESDAY.

Meeting called to order at 10 o'clock A. M. by President Lathrop. Minutes read and approved.

*Dr. N. S. Hoff*, read a paper entitled, "Systematic Medication."

The caption of this paper implies that, in our mind at least, there is such a thing as unsystematic or careless medication. The evidence may be had by noting the methods of our neighbors, journal articles, cases which fall into our hands from other practitioners, or a visit to the dental offices of the profession, and an inspection of their medicine chests, noting the medicines generally found and their condition. In some cases quite a collection of bottles containing various remedies; usually a large percentage will be compounds made after some published formula that the author used successfully, but we find this bottle unused —indicated by the dust which covers it—because the formula was not made to cure every thing from tooth ache to necrosis, and is therefore set aside for the more reliable creosote or carbolic acid bottles which have a prominent place on the front shelf and are evidently much patronized.

In the majority of offices the armamentarium will be found to consist of carbolic acid, creosote, tincture of iodine, tincture of aconite, arsenious acid, aqua ammonia, alcohol, tannic acid, etc. This is not such a bad equipment and possibly it may be all the owner's intelligence requires, but it is not much in advance of the dental outfit of forty years ago. It is not the meagreness or faulty selection of remedies that we complain of so much as their empirical use. For an intelligent use of the list given above will equip an average practitioner to cope with the requrirements of his business.

It ought to be a matter of concern to us all that in this day of progress and enlightenment, because of the universal dissemination of knowledge through the dental periodical literature and accessible dental societies, text books, etc., that more progress has not been made in the scientific application of medicines to the cure of dental diseases. It would seem that all the newer remedies, especially the approved ones, should be in the hands of the profession, not only that the patient may have the benefit of their use, but that by a wider use, sufficient and accurate data might be quickly secured as to the action of these remedies in the treatment of diseases of the dental tissues.

It is the careless use (we trust it may not be due to ignorance) of dental remedies that convinces us that our medication is, as it is frequently practiced, empirical and unsystematic, and consequently oftentimes tedious, and inefficient if not harmful.

There seems to exist the same lack of uniformity as to methods of procedure in the treatment of diseases of the dental organs, as in the methods of operating for their preservation by filling. And it is highly probable that the reasons in one case are the same as those which obtain in the other. The methods of filling teeth have not been evolved from the brain of the scientist or from his laboratory; but they have come with years of practice and experience from the men who have by their indomitable energy and perseverance amidst business perplexities, conceived the ideas, frequently worthless if not injurious, generally crude; and which it was necessary always to submit to the tedious and expensive—to both patient and dentist—practical tests of unscientific experimentation and development.

To-day our conceptions are scrutinized by scientific dentists, for our profession is now blessed with such men, and we are saved the long and tedious experimentation involved in the practical tests of former years. While this is particularly true in regard to the more practical affairs, it is also true that our scientific dentists are at work upon causes and conditions of disease. Physiological and pathological dental tissues, and the action of reagents upon such structures, are better understood now than ever before, and the fact that no scientific classification of dental drugs and diseases is known to or used by the mass of the profession, is undoubtedly due to the fact that the profession has not kept pace with the advance of its own and other kindred sciences.

To illustrate the point we wish to make, we will take the liberty of referring to the drugs most commonly used by dentists. The old-time creosote and latterly carbolic acid, have retained their hold upon the dental profession thus long, not because of any particular virtue either one possesses, but because of their combination of properties. Carbolic acid is a stimulant, coagulant, escharotic, germicide, and antiseptic. It may be compared to a shot gun load, as it meets so many requirements, some one or more of which are generally present in every case requiring medication; but like the shot gun load it does not go singly and directly to the mark as a rifle ball does, but may scatter and destroy or mutilate other structures that it is exceedingly desirable to preserve. This agent undoubtedly has been a great boon to the profession and many cures have resulted from its use, but too often at great cost to other structures has this desirable result been obtained.

So long as we were working as it were in a very dim light in regard to the nature of disease, this agent was a great boon, but now that we know definitely what inflammation is, what causes it, and exactly how it takes place, and what the progressive stages are, and can also foresee results, it does seem that we ought to begin to apply remedies directly and definitely. It has been satisfactorily demonstrated to us that we cannot have suppuration without the presence of a micro-organism; and we have also been shown that certain agents are hostile to the micro-organism and some are capable of completely destroying them; and consequently with our limited but distorted vision we see no necessity for the application of any remedial agent other than a germicide—under some circumstances the worst possible agent that could be employed. This practice is no less wrong and unsystematic than to apply the one agent as a cure all in every case. Such a proceeding is about as systematic and scientific as it would be to insert an artificial denture in a mouth full of diseased, loosened and tartar covered teeth, and expect a healthy and satisfactory healthy process to take place, simply because we have used a material upon which to construct the denture, that experience has taught us is likely to subdue inflammatory conditions of the mucous membrane, and which we hope, because of careful adoption and skillful construction, will bring about the desired healthy condition. Just as certainly as some preliminary work is demanded in this illustrative case, so certainly is there generally a necessity for preliminary work to that of medication. In the treatment of diseases of pulps, alveolar abscess, pyorrhœa alveolaris, etc., some things are needed to be done before any medication will be beneficial. The pulp must be freed from all foreign and encumbering substances, the abscess must be so opened as to secure free and certain drainage, the salivary calculus must be removed and the teeth ligatured together if loose; many other simple and mechanical details must be attended to before any medication can be successfully applied. Douches or injections of warm sterilized water should follow the surgical means, in order that all fragments loosened by the instruments, as well as the blood and pus contained in the tissues which might be coagulated by some of the medical detergents, should be removed, as such a coagulum would remain as an irritant and hinderance to the direct and free application of subsequent medical agents to the diseased structures.

The next agent to be applied is a disinfectant. A combination deodorizer and germicide can usually be applied, as most germicides are more or less deodorizers. The parts are then ready for an antiseptic dressing and a period of rest. An antiseptic should be selected that will not become decomposed or dissipated too quickly, but by its slow decomposition and liberation of its sterilizing agent, will remain for some time in contact with the infected tissues, preventing the development and growth of microorganisms, and giving time for the enfeebled tissues to take on a more healthy function. If the tissues show no disposition to heal under this mechanical and antiseptic treatment, supposing it to have been thoroughly and carefully done, the indications then would be for the application of curative agents locally, stimulants or counter-irritants, systemically, tonics, alteratives, etc. Just at this point there is an excellent opportunity for the dentist to display his judgment if he is happy enough to have any that is based on medical science. Not that it may be necessary to supplant the physician, but to advise with him and ask his counsel and help in correcting systemic disorders, which very frequently are of sufficient force to thwart all well directed efforts for the successful treatment of local disturbances, although the active cause may be local in its nature.

Frequently it becomes necessary to destroy or remove a portion of the tissues which have become so enfeebled, that they will not take on again the normal function. For this, surgical treatment is generally preferable, but frequently it is not practi cable, and we are forced to resort to the escharotics, painful and difficult to control though they may be. This treatment must be followed by the same treatment indicated above; detergents, disinfectants and antiseptics, accompanied by astringents or stimulants when indicated. A successful issue may be confidently predicted on the basis of science and clinical procedure if the details of the above outline are carefully considered. The point that we want to emphasize is, that agents of a single and definite action, when applied systematically may be expected to accomplish better results, quicker and with less destruction of valuable tissues, than those possessing numerous virtues, some of which it is not desirable to exhibit in peculiar conditions, because of the possibility of producing more harmful than beneficial results.

To illustrate, if possible more vividly, the systematic application of one class of approved remedies in the treatment of diseased dental structures, I would like to make the following tabular classification for use in the treatment of dental and oral diseases where suppuration is present. I would give preference to the first named agent in each class.

| | | |
|---|---|---|
| DETERGENTS, | 1. Manipulative measures.<br>2. Warm sterilized water. | Both usually. |
| DEODORIZERS, | 1. Permanganate of potassium.<br>2. Peroxide of hydrogen.<br>3. Lysterine. | |
| GERMICIDES, | 1. Bichloride of mercury.<br>2. Peroxide of hydrogen.<br>3. Carbolic acid. | |
| ANTISEPTICS, | 1. Aristol.<br>2. Black's 1, 2, 3 remedy.<br>3. Boracic acid. | |
| STIMULANTS, | 1. Alcohol and its tinctures.<br>2. Aromatic sulphuric acid.<br>3. Oil of cloves and sulphate of copper when it is desirable to have an astringent effect. | |
| ESCHAROTICS, | 1. Carbolic acid.<br>2. Mineral acids.<br>3. Arsenious acid and potassium hydrate. | |

DISCUSSION.

*Dr. J. Taft.*—Sterilized warm water is a most excellent detergent, but it is in many cases insufficient. I have used the warm water to which a small quantity of soap had been added. If some alkali, such as the bicarbonate of soda, was added to the water the solvent property of the water would be increased without danger of introducing any harmful agent. I use pepsin with satisfaction in the treatment of exposed pulps that are encumbered with much disorganized tooth structure or other debris; it has a very good and satisfactory action.

*Dr. C. S. Case.*—I don't think warm water as a detergent can be compared with peroxide of hydrogen, which by its evanescence throws out mechanically much foreign material and thereby cleanses the cavity, while the elements liberated by its decomposition, break down and render soluble the insoluble materials contained in alveolar cysts, etc.

*Dr. Hoff.*—The peroxide of hydrogen that is placed upon the market is not free from acid, and its use in the treatment of teeth having foul pulps or alveolar abscesses through the pulp canals is not desirable, because of its solvent effect upon the hard tissues of the root. Then again, it is capable of coagulating pus in a closed pocket, where it will remain for some time as an irritant before it can be dissolved and washed away. Consequently the use of peroxide of hydrogen should always be preceded by sterilized warm water to free the cavity from coagulable material.

Adjourned to meet at 2 P. M.

The association was called to order at 2 P. M., and Prof. C. S. Case, in lieu of a paper, gave a lecture on Correcting Irregularities of the Teeth.

When the majority of dentists undertake to regulate a set of teeth they immediately devise and construct a plate with springs and other attachments which are inconvenient for the patient to wear and which I think are entirely unnecessary. With a plate there is no definiteness as to the extent of the movement secured and its action is usually continuous, allowing no time for rest and recuperation. The plate is also more or less uncleanly. I use the "coffin plates" for spreading the arch, but never for moving teeth. I prefer an appliance with which exact and definite movements may be secured and periods of rest may be secured. I also want to know what amount of pressure or force I am exerting. Exactness of movement can not be obtained by the use of rubber bands, springs and ligatures. These springs and bands, because of their continuous force, are capable of doing great harm. The changes which the tissues are compelled to undergo in the process of regulating teeth, are not sufficiently understood. More attention should be given to this matter; our text books do not give it the consideration it deserves, and it should be taught more thoroughly in the colleges.

The "Angle" system of regulating, by means of screws, so arranged as to produce the desired movements with certainty and exactness, by manipulating at intervals the screws, is the most definite and scientific method of changing the position of teeth known.

The doctor here described the methods of constructing the different kinds of jack-screws, etc., for use with this system, exhibiting a great many models with appliances in place, showing how the different forms of irregularities could be satisfactorily corrected by this method. The appliances are all made of German silver, except the nuts for the screws which are cut from five cent nickel coin. For bands No. 16 German silver wire is rolled down nearly as thin as it is practicable to roll it on an ordinary mill; considerably thinner than No. 36 plate gauge. Its toughness and high fusing power admirably adapt it for easily fitting and soldering, and even though very thin it will have sufficient strength to resist the force of any traction to which it may be subjected. The bars upon which the thread is cut are made of German silver wire, the size varying with the ideas and convenience of the manufacturer. The doctor uses No. 18 wire and draws it down somewhat in order to temper or stiffen it, so that it is large enough to cut the thread with No. 6 hole of the Martin twin hole screw-plate. From this hole in the screw-plate a thread is cut on a piece of softened steel wire to make a tap for cutting the thread in the nut. The point of this tap is then dressed to a triangular point and tempered. Holes of the proper size are drilled into the nickel coin and the tap run through them, cutting the thread. The coin is then sawed into little square blocks making a nut which can afterwards be filed to any desired size or shape so as to fit the wrench used in manipulating them. Considerable care is required to make all these things accurately, so that when the appliance is made and placed in position its movement will be positive and easy. The tubing into which the bars fit is made by cutting strips of German silver plate, No. 28 plate gauge, about one-fourth inch wide, and after turning up the edges by stamping into it a round excavator handle to expedite its passing, it is drawn through a wire draw plate, successively through the largest holes first, down to the one of proper size, when it will be found that a nice piece of tubing into which the jack-screw will exactly fit has been obtained. This can then be cut into such lengths as the case may require. These jack-screws can be made to pull or push, and one screw can be made by adding attachments to its tube, or the nut, or the screw itself, to exert force in four or five different directions, while the screw is only turned one way. The versatility of the system can not be fully appreciated unless one sees the application to models in which almost every variety of irregularity has been corrected. The appliance may be gold plated after it is made if desired, but this is not necessary as the German silver does not corrode, although it tarnishes in the mouth.

*Dr. Parker*, of Grand Rapids, suggested that when tempering the steel taps yellow, prussiate of potassium mixed with flour and water so as to make a paste, would be an excellent mixture into which the steel tap should be plunged before it is heated. It prevents the steel from scaling and thus destroying the edges of the delicate screw threads.

*Dr. H. H. Jackson*, of Detroit, exhibited a number of articulated plaster models showing the manner in which cases of irregularity had been treated by means of springs made of piano wire and attached to the teeth by means of wire cribs, dispensing with all plates. He also gave a clinic illustrating the method of making the cribs and springs. The method is the same as that advocated by Dr. V. H. Jackson, of New York city, and referred to in the report of the proceedings of the thirty-first annual meeting of the American Dental Association on pages 541 and 542 of the Dental Register for November, 1891.

*Dr. Hoff* exhibited a model with a plate, which was used by Dr. Bodecker, of New York city, to draw back the upper incisors, which were very much protruded, and at the same time expand the arch which was contracted to a v shape. A rubber plate is made to cover the vault of the jaw, into this a flat gold band is vulcanized, passing out between the second bicuspid and first molar, and thence upon the labial faces of the teeth around to the corresponding place on the opposite side of the arch where it passes between the teeth, and is vulcanized into the rubber plate. The rubber plate is sawed on both sides of the palatal ridge three-fourths of the distance, beginning between the lateral incisor and cuspid, toward the posterior edge of the plate, on a line midway between the median ridge of the hard palate and alveolar process. A Talbott wire spring is then bent to conform to the plate and the ends inserted into the splint plate opposite the cuspid teeth, while the coil of the spring lies in the median line and near the posterior edge of the plate. The spreading of spring causes the plate to expand and this carries outward the cuspid and bicuspid teeth causing the widening of the arch, at the same time the spreading of the arch puts a tension on the gold band which draws the incisors backward. Dr. Bodecker claims this is a very effective apparatus for the purpose.

EVENING SESSION.

At the evening session Dr. Hoff exhibited a number of new appliances and instruments secured through the kindness of the manufacturers and dealers. Including Dr. Timme's method of making glass inlays, a new base plate material, a new form of crystal gold, flexo separating slips, fissure burs, electric root dryers, electric gutta-percha filling packer, composition cement and asbestos filling material, sand paper strips, disk mandrels, etc., etc. He also exhibited a case of specimens illustrating a method of making a compound filling of cement and gold. The larger part of the cavity is filled with cement and while it is yet soft a little cake made of gold foil, approximating in form and size the cavity, is forced into it and the cement quickly set by injecting warm air into it. This little cake of foil retains a good receiving surface for gold foil with which the operation can be completed in the ordinary way. The idea comes from Germany and is interesting more because of the earnest endeavor of its author to do something for his profession, than that it will ever be extensively adopted. But it is possible this idea, in some modification, may at times be a very acceptable method for overcoming a perplexing operation.

There being no further papers to be read it was voted to elect officers and adjourn this evening.

The officers elected were: President, H. C. Corns, Detroit; First Vice-President, G. E. Sanders, Saginaw; Second Vice-President, N. S. Hoff, Ann Arbor; Secretary, J. Ward House, Grand Rapids; Treasurer, G. H. Mosher, Jackson.

Executive Committee: L. F. Owen, Grand Rapids; E. C. Moore, Detroit; Wm. Cleland, Detroit.

Local Committee of Arrangements: G. E. Saunders, Saginaw. R. P. Alden, Saginaw.

Supervisor of Clinics: W. A. Dorland, Grand Rapids.

Board of Censors: J. W. House, Grand Rapids; J. L. Gish, Jackson; J. L. Sweetnam, Manistee.

University Visiting Committee: J. B. McGregor, Port Huron; J. Lathrop, Detroit; J. A. Harris, Pontiac; G. L. Field, Detroit; H. H. Jackson, Detroit.

Legislative Committee: A. T. Metcalf, Kalamazoo; W. H. Dorrance, Ann Arbor.

The treasurer reported a balance on hand of $187.09 after paying all bills. The association voted twenty-five dollars to the American Dental Association to assist in carrying on the work of examining the prehistoric crania of the country,

It was voted to hold the next meeting at Saginaw, June 7th, 1892. A vote of thanks was extended to Drs. Conway and Williams for the eminently satisfactory way in which they had provided for the comfort and convenience of the members during the meeting.

Adjourned.

